# Tailoring Blue-Green Double Emissions in Carbon Quantum Dots via Co-Doping Engineering by Competition Mechanism between Chlorine-Related States and Conjugated π-Domains

**DOI:** 10.3390/nano8090635

**Published:** 2018-08-21

**Authors:** Xue Sun, Huilian Liu, Lili Yang, Xinying Wang, Weiqiang Yang, Maobin Wei, Xiaoyan Liu, Jian Cao, Jinghai Yang, Scott Guozhong Xing

**Affiliations:** 1 Key Laboratory of Functional Materials Physics and Chemistry of the Ministry of Education, Jilin Normal University, Changchun 130013, China; xuesun1992@126.com (X.S.); jlsdzccw@126.com (M.W.); liuxiaoyan1437@163.com (X.L.); caojian_928@163.com (J.C.); jhyang1@jlnu.edu.cn (J.Y.); 2 National Demonstration Center for Experimental Physics Education, Jilin Normal University, Siping 136000, China; 3 School of Engineering and Architecture, Northeast Electric Power University, Jilin 132012, China; wangxinying.2008@163.com; 4 Centre for Advanced Optoelectronic Functional Materials Research and Key Laboratory of UV-Emitting Materials and Technology (Northeast Normal University), Ministry of Education, Changchun 130024, China; yangweiqiang123456@163.com; 5 United Microelect Corp. Ltd., 3 Pasir Ris Dr 12, Singapore 519528, Singapore

**Keywords:** carbon quantum dots, co-doping, competition mechanism, conjugated π-domain

## Abstract

Representing single-layer to tens of layers of graphene in a size less than 30 nm, carbon quantum dots (CQDs) is becoming an advanced multifunctional material for its unique optical, electronic, spin and photoelectric properties induced by the quantum confinement effect and edge effect. In present work, upon co-doping engineering, nitrogen and chlorine co-doped CQDs with uniquely strong blue-green double emissions are developed via a facile and one-pot hydrothermal method. The crystalline and optical properties of CQDs have been well manipulated by tuning the mole ratio of nitrogen/chlorine and the reaction time. The characteristic green emission centered at 512 nm has been verified, originating from the chlorine-related states, the other blue emissions centered at 460 nm are attributed to the conjugated π-domain. Increasing the proportion of 1,2,4-benzentriamine dihydrochloride can effectively adjust the bandgap of CQDs, mainly caused by the synergy and competition of chlorine-related states and the conjugated π-domain. Prolonging the reaction time promotes more nitrogen and chlorine dopants incorporate into CQDs, which inhibits the growth of CQDs to reduce the average size of CQDs down to 1.5 nm, so that the quantum confinement effect dominates into play. This work not only provides a candidate with excellent optical properties for heteroatoms-doped carbon materials but also benefits to stimulate the intensive studies for co-doped carbon with chlorine as one of new dopants paradigm.

## 1. Introduction

Nanocrystals have received both fundamental and practical attention owing to their potential applications in many fields such as ultrasensitive biosensing, imaging agents, photothermal therapy and catalysts, due to their tunable optical properties and high catalytic activities [1,2,3,4,5,6,7,8,9]. Carbon quantum dots (CQDs) are regarded as an emerging functional materials due to their great advantages such as biocompatibility, low toxicity, simple preparation technique and high photostability [10,11,12,13,14,15,16,17,18], which have been widely applied in the fields of optical detection probes, light-emitting diode, photocatalysis and so on [15,16,17]. Doping heteroatom into CQDs is an effective technique to endow new or improved photoluminescence properties and extend their application potential.

So far, the elements such as S, N, B, Na, Se and Cl have been served as dopant in CQDs to exhibit distinct photoluminescence properties and the corresponding origination of emission has been discussed in detail [17,18,19,20]. For instance, Yang et al. demonstrate that N and Se doping can achieve a tunable emission wavelength in CQDs and the fluorescence is close to the surface activity groups [21]; Qu et al. prepared S, N co-doped CQDs with multicolor emissions and found that the luminescence may come from the carbon core [18]. Obviously, the above works not only realized the tunable emission in CQDs via heteroatom doping but proposed the emission origination. Normally, the emission origination of CQDs can be divided into two categories: one is intrinsic state related to conjugated π-domains in CQDs [22,23], the other is surface/defect/edge state related to the surface groups or defects [24,25,26,27]. However, carbon cores, surface states and defect states usually coexist in the as-prepared CQDs and all of them contribute to the photoluminescence process. Unfortunately, the synergy or competition mechanism between intrinsic states and surface/defect states is difficult to be revealed due to its complex characteristic, especially the quantum confinement effect is introduced into the CQDs series at the same time, which needs pay more efforts for the researchers to explore the detailed mechanism.

Chlorine was introduced into carbon materials for the first time by Yang group at 2013 [28]. They realized the multicolor emission in chlorine-doped CQDs and attributed such novel optical properties to the additional energy levels between C π and C π* introduced by Cl dopants. Zhao et al. [29] then fabricated the chlorine doped CQDs by way of the chemical exfoliation of HCl treated carbon fibers and the related photovoltaic detectors exhibited an exceptionally big ratio of photocurrent to dark current as high as ~105. Although chlorine doping can bring excellent application performance, the relative research works are rather rare due to the big radius mismatch between Cl and C, especially for double heteroatoms co-doping. The investigation about double-heteroatom doping with Cl as one kind of dopant in CQDs is almost blank. Therefore, we aim to achieve the nitrogen and chlorine co-doped CQDs to tune the emission wavelength and further investigate co-doping effects on the luminescent properties of CQDs. Moreover, the luminescence mechanism of the CQDs is much vaguer after element doping, especially for co-doping CQDs, which means that the PL mechanism of CQDs is still an open topic. The detailed synergy and competition mechanism of carbon cores and surface/defect states during the photoluminescence process in such kind of CQDs is necessary to be revealed, which is beneficial for extending the application field and enhancing the application performance of CQDs.

In present work, nitrogen and chlorine co-doped CQDs with unique strong blue-green emission were synthesized through a facile and one-pot hydrothermal method with 1,2,4-benzentriamine dihydrochloride (BD) as nitrogen and chlorine sources. Crystalline and optical properties of CQDs have been well tuned by controlling the mole ratio of citric acid to BD and reaction time. The detailed synergy and competition of chlorine-related states and conjugated π-domain via gradually varied concentration of nitrogen and chlorine dopants has been discussed in detail.

## 2. Experimental

### 2.1. Materials 

All the chemicals were utilized without any further purification. Monohydrate citric acid (CA) was purchased from Beijing Chemical Works (Beijing, China). 1,2,4-Benzeneetriamine dihydrochloride (BD) was purchased from Energy Chemical (Shanghai, China), the deionized water was purified using a Millipore (Shanghai, China) system throughout all experiments.

### 2.2. Synthesis of N,Cl Co-Doped CQDs

In comparison with counterpart bulk composites, the nanostructured materials equipped with large surface area demonstrate the superior optical, magnetic and electrical characteristics [28,29,30,31,32,33,34,35,36,37,38,39,40,41]. The N,Cl co-doped CQDs were prepared by a novel and one-pot hydrothermal method: The certain quantity of CA and BD were dissolved in 10 mL deionized water with well-stirring to form a transparent solution. Thereafter, the transparent solution was transferred into a 50 mL Teflon-lined stainless-steel autoclave. The autoclave was kept in an electric oven under 200 °C with different duration time. The collected CQDs were dialyzed for 24 h in deionized water with the molecular weight cut-off (MWCO) dialysis bag of 3000 Da and then further dialyzed for 12 h in deionized water using a MWCO of 3500 Da. Finally, vacuum-rotary evaporation was performed to remove the water. The obtained CQDs powder can be easily redispersed in water with a certain concentration to set aside for subsequent characterization. In this work, two sets of CQDs were synthesized. One set was prepared by varying the reactant molar concentration ratio of CA and BD for 1:0.5, 1:1, 1:1.5 and 1:2 for reacting 24 h. CA was added at 0.4204 g (2 mmol) and 0.1961 g (1 mmol), 0.3923 g (2 mmol), 0.5884 g (3 mmol) and 0.7845 g (4 mmol) of BD was added, respectively. The corresponding samples were then names as CQDs (1:0.5), CQDs (1:1), CQDs (1:1.5) and CQDs (1:2), respectively. The other set of samples was prepared by prolonging the reaction duration for 6 h, 12 h, 24 h, 36 h or 48 h with maintaining the reactant molar concentration ratio of CA and BD for 1:1. The corresponding samples were then names as CQDs (6 h), CQDs (12 h), CQDs (24 h), CQDs (36 h) and CQDs (48 h), respectively. 

**Figure 1:**
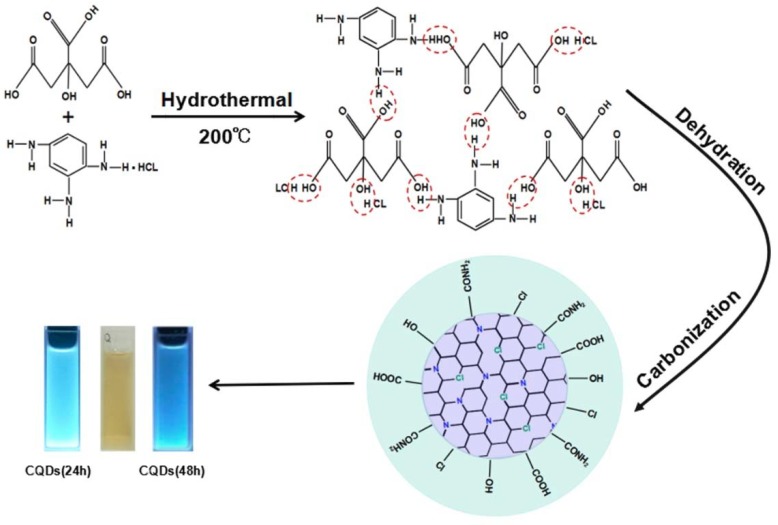
The scheme image of synthetic process of nitrogen and chlorine co-doped carbon quantum dots (CQDs).

The scheme image of synthetic process of CQDs in our case is shown in Figure 1. Under the hydrothermal conditions, CA and BD undergoes dehydration. During this process, hydrochloric acid will dissociate H^+^, so that chlorine will enter the CQDs in one step. Meanwhile, we discovered that an N atom enters CQDs by forming a pyrrolic structure through intramolecular dehydroxylation between neighboring carboxyl groups. Pyrrolic N is gradually transferred into quaternary N in the graphene with increasing the reaction time [42].

### 2.3. Characterization

Transmission electron microscope (TEM) and high-resolution TEM images were performed with JEM-2100 electron microscope (JEOL Ltd., Tokyo, Japan) at an operating voltage of 200 kV. The atomic force microscopy (AFM) images were obtained on an Agilent 5500 SPM (Agilent Technologies Inc., Santa Clara, CA, USA) in AC mode with a silicon probe (NT-MDT, cantilever force constant 40 N m^−1^). Dynamic light scattering (DLS) was detected using a Nano-ZS (Malvern Instruments, Malvern, UK). Raman spectra were obtained from an inVia™ confocal Raman microscope of Renishaw with 514 nm wavelength excitation laser (Renishaw, London, UK). The X-ray photoelectron spectroscopy (XPS) was performed using an Al Kα monochromatized source (Thermo Fisher Scientific, Waltham, MA, USA) to acquire elemental information. Fourier-transform infrared (FT-IR) spectra of CQDs were recorded using KBr pellets with a Bruker Vertex 70 spectrometer (Bruker, Billerica, Massachusetts, US) from 4000 to 400 cm^−1^. The ultraviolet-visible absorption spectra were obtained by UV-3600PC UV-Vis spectrophotometer of SHIMADZU Corp. (Shimadzu, Tokyo, Japan). The photoluminescence spectrum was recorded on a FL-1000 luminescence spectrometer (iHR320, Horiba Jobin Yvon, Paris, France) at room temperature in an aqueous solution. Time-resolved PL behavior was measured via the time-correlated single-photon counting (TCSPC) technique (Horiba Jobin Yvon, Paris, France).

## 3. Results and Discussion

### 3.1. Effects of Reagent Ratio

**Figure 2:**
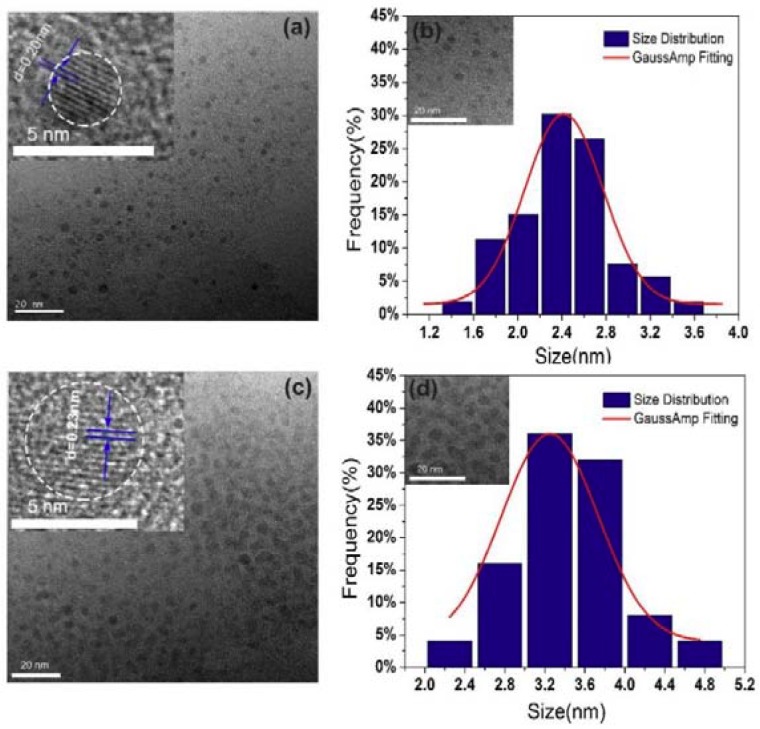
(**a**,**c**) Transmission electron microscopy (TEM) and high-resolution TEM (HRTEM) (insert) images of CQDs (1:1) and CQDs (1:2); (**b**,**d**) The size distribution and local-enlarged TEM image (inset) of the CQDs (1:1) and CQDs (1:2).

From dedicated structural characterization perspectives, TEM measurement is an effective methodology for structure research in the materials science and materials engineering [43,44,45,46,47,48,49,50,51,52,53,54,55,56]. The TEM technique was further utilized to characterize the microstructure and particle size of CQDs. The TEM images of CQDs (1:1) and CQDs (1:2) are shown in Figure 2a,c. Both as-prepared CQDs exhibit uniform dispersion without apparent aggregation. The HR-TEM images of CQDs (1:1) and CQDs (1:2) (insets in Figure 2a,c) illustrate the distance between the lattice fringes is about 0.20 nm and 0.23 nm, respectively, which is close to (100) facet of graphite [57,58]. Figure 2b,d illustrates the particle size distribution of CQDs (1:1) and CQDs (1:2). The mean diameter can be determined to be 2.5 nm and 3.3 nm, respectively. Obviously, the particle size of CQDs (1:2) is enlarged with the increase of BD amount and the distance between particles is much closer than that of CQDs (1:1) as shown in the insets of Figure 2b,d. During the hydrothermal process, in comparison with CQDs (1:1), the increase of π-conjugate area due to the increase of the BD amount leads to the size enlargement of CQDs (1:2). Meanwhile, due to the amount of benzene rings and chlorine increased simultaneously, chlorine and nitrogen got more opportunity to enter the carbon core, so that the lattice fringes increase accordingly. 

**Figure 3:**
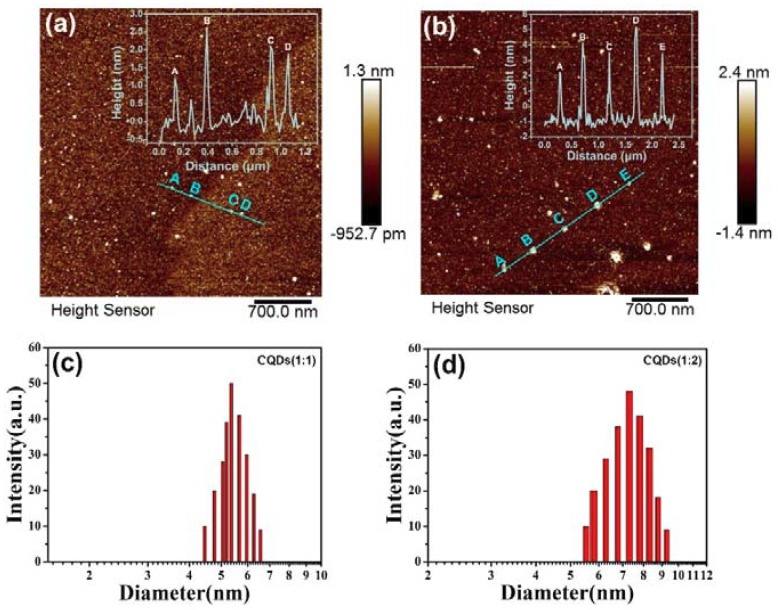
(**a**,**b**) Atomic force microscopy (AFM) images of CQDs (1:1) and CQDs (1:2) deposited on a silicon slice (inset: the height profile along the line in the topographic image of the CQDs (1:1) and CQDs (1:2), respectively); (**c**,**d**) Dynamic light scattering (DLS) images of CQDs (1:1) and CQDs (1:2).

The atomic force microscopy (AFM) and Dynamic light scattering (DLS) techniques were further demonstrates the topographic morphology and particle distribution of the CQDs. The corresponding AFM images are shown in Figure 3. The typical topographic height of CQDs (1:1) and CQDs (1:2) is ca. 0.5–2.5 nm and 2–5 nm, respectively, suggesting that the CQDs (1:1) consists of ca. 1–5 graphene layers and CQDs (1:2) consists of 4–10 graphene layers, which is also consistent with previous reports [59,60,61,62]. DLS further characterizes the distribution of CQDs in aqueous solution. The average particle size of the CQDs (1:1) and CQDs (1:2) is 5.47 nm and 7.3 nm, respectively. DLS measurements indicated that the average particle size of the CQDs nanoparticles become a little bigger than that of the TEM characterization, which is reasonable since DLS characterizes the particle size of the CQDs in the hydrated state. Thus, we can confirm that the as-prepared CQDs in our case are carbon quantum dots.

**Figure 4:**
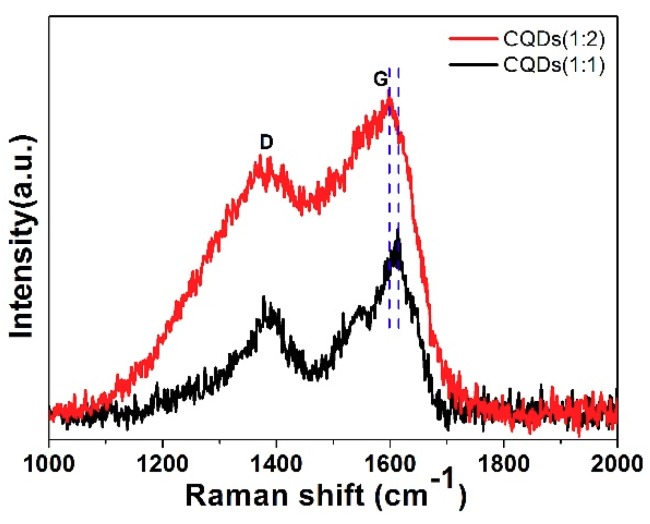
Raman spectra (λ_ex_ = 514 nm) of CQDs (1:1) and CQDs (1:2).

Raman spectroscopy was used to further demonstrate the intrinsic structure of CQDs. The Raman spectra of CQDs (1:1) and CQDs (1:2) are shown in Figure 4. Both Raman spectra of samples exhibit two Raman peaks: one peak located at ~1360 cm^−1^ can be ascribed to defect-related D-band, the other peak located at ~1580 cm^−1^ can be ascribed to G-band related to sp^2^ carbon networks. The intensity ratio of D and G band (I_D_/I_G_) is usually used to represent the degree of carbonization and defects of CQDs and the larger value of I_D_/I_G_ indicates the low degree of carbonization and more defects [63]. Gu et al. reported that doping heteroatom to CQDs can introduce more defects in the graphene lattice and change the lattice fringes of CQDs [64]. In our case, the particle size and lattice fringes of CQDs (1:2) increased compared to CQDs (1:1) and the value of I_D_/I_G_ for CQDs (1:1) and CQDs (1:2) is calculated to be 0.52 and 0.78, respectively. Thus, the increased I_D_/I_G_ ratio for CQDs (1:2) can be attributed to the appearance of more defects caused by introducing more nitrogen and chlorine into CQDs (1:2). Moreover, compared to the Raman spectrum of CQDs (1:1), the peak position of the D-band in CQDs (1:2) kept stay, while the position of G-band shifted to lower wavenumber by 10 cm^−1^, which can be attributed to the phonon stiffening in CQDs (1:2) [65,66]. 

**Figure 5:**
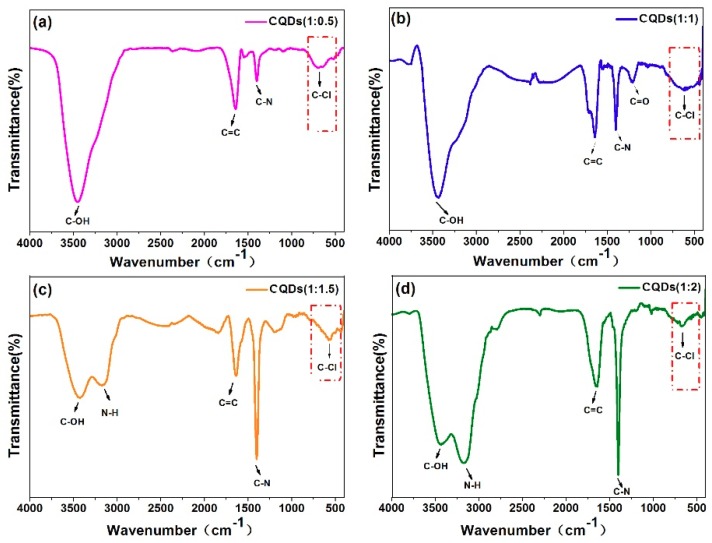
Fourier transform infrared (FTIR) spectra of CQDs (1:0.5) (**a**), CQDs (1:1) (**b**), CQDs (1:1.5) (**c**) and CQDs (1:2) (**d**).

The information of surface functional group can be obtained by Fourier transform infrared (FTIR) spectra. Figure 5 illustrated the FTIR spectra of CQDs (1:0.5), CQDs (1:1), CQDs (1:1.5) and CQDs (1:2). All FTIR spectra demonstrate two strong absorption band located at 1640 cm^−1^ and 1400 cm^−1^, which can be ascribed to the strong stretching vibrations of C=C and C–N, respectively. Meanwhile, broad absorption bands at 3000–3600 cm^−1^ can be attributed to C–OH and N–H stretching vibrations, respectively. It is interesting to observe another absorption band located at 600–800 cm^−1^, which can be assigned to C–Cl vibration mode, indicating chlorine has been successfully doped into CQDs [19,67,68].

**Figure 6:**
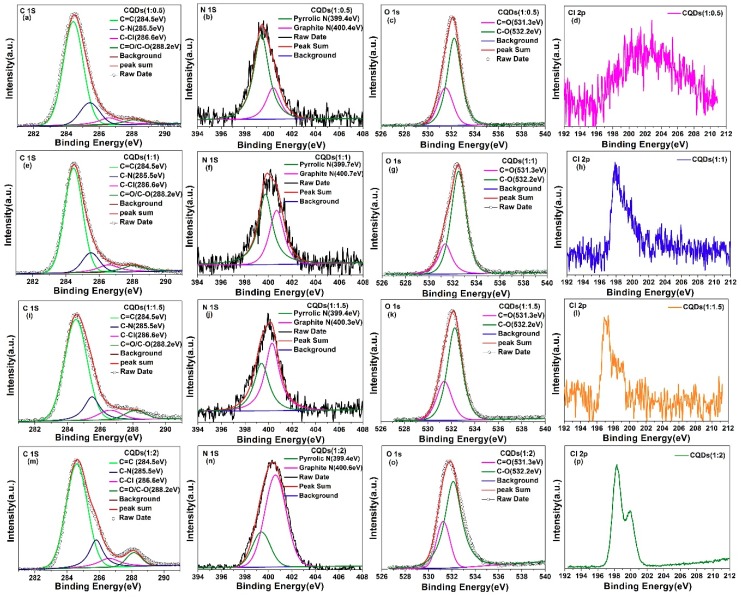
High-resolution C 1s, N 1s, O 1s and Cl 2p core level X-ray photoelectron spectroscopy (XPS) spectra of CQDs (1:0.5) (**a**–**d**), CQDs (1:1) (**e**–**h**), CQDs (1:1.5) (**i**–**l**) and CQDs (1:2) (**m**–**p**).

X-ray photoelectron spectroscopy (XPS) was further utilized to obtain the functional group and chemical composition information of CQDs. Figure 6 presented the high-resolution C 1s, N 1s, O 1s and Cl 2p core levels XPS spectra of CQDs (1:0.5), CQDs (1:1), CQDs (1:1.5) and CQDs (1:2) samples. As shown in Figure 6a,e,i,m, four peaks are necessary for well-fitting C 1s XPS spectra, indicating four carbon species exist in CQDs samples. The fitting peaks are located at 284.5 eV and 288.2 eV, which can be attributed to C=C and C=O/C–O bond, respectively [28,69,70,71]. Meanwhile, another two fitting peaks are located at 285.5 eV and 286.6 eV, which can be assigned to C–N and C–Cl bond [72,73], respectively. It is consistent with the results of FTIR, which further proves nitrogen and chlorine have been successfully introduced into CQDs. With varying the ratio of CA:BD from 1:0.5 to 1:2, the content of chlorine and nitrogen continuously increased and reach to the maximum when the ratio of CA and BD is 1:2. The high-resolution O 1s spectra are shown in Figure 6c,g,k,o. The two fitted peaks at 531.3 and 532.4 eV are ascribed to C=O and C–O groups [74,75], respectively. The existence of C–O and C=O bonds indicated that the surface of the as-synthesized CQDs is functionalized by multiple oxygenated groups, which is consistent with C1s spectra. The C/O atomic ratio for the CQDs (1:0.5), CQDs (1:1), CQDs (1:1.5) and CQDs (1:2) is 1.34, 1.49, 1.89 and 3.2, respectively. The increased C/O ratio is related to the amount of BD. With increasing BD from 0.5 mmol to 2 mmol, the content of carbon increased at the same time, so that the C/O becomes larger. To testify the continuously increase of Cl and N doping concentration, the high-resolution N 1s and Cl 2p core level XPS spectra of CQDs (1:0.5), CQDs (1:1), CQDs (1:1.5) and CQDs (1:2) samples are shown in Figure 6b,f,j,n and Figure 6d,h,l,p. The binding energy located at ~399.4 eV and ~400.4 eV can be attributed to pyrrolic N and graphitic N, respectively. The binding energy located at 198 eV and 202 eV can be assigned to Cl 2p^1/2^ and Cl 2p^3/2^ core lines, respectively. Obviously, with varying the ratio of CA:BD from 1:0.5 to 1:2, the content of graphitic N and the signal intensity of Cl 2p continuously increases and the peak shape of Cl 2p gradually turns better, indicating that the doping concentration of chlorine increases. The percentages of C–N and C–Cl bond of four samples are listed in Table 1.

Taking account to the results obtained from TEM, Raman, XPS and FTIR spectra, two important facts for CQDs in our case can be extracted. The N, Cl co-doped CQDs can be successfully synthesized with much higher degree of carbonization compared with other reports [76,77,78,79] and the nitrogen and chlorine doping concentration can be well controlled by adjusting the precursor ratio of CA:BD. Secondly, the existence of C–Cl and C–N bond and its corresponding concentration in CQDs exhibited great influence on the degree of carbonization of CQDs, implying that the introduction of nitrogen and chlorine might significantly affect the luminescent properties of CQDs.

**Figure 7:**
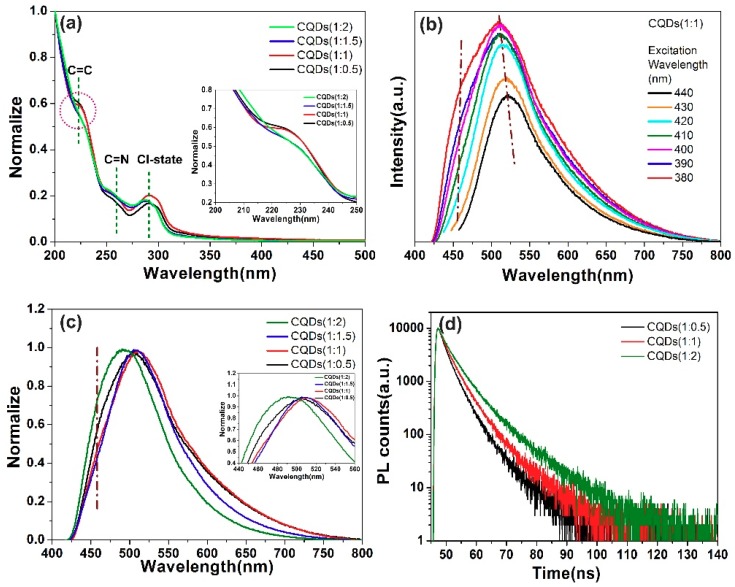
(**a**) UV-Vis absorption spectra of CQDs (1:0.5), CQDs (1:1), CQDs (1:1.5) and CQDs (1:2) in aqueous solutions (The inset is an enlarged view of absorbed by C=C); (**b**) PL spectra of CQDs (1:1) excited at wavelength of 380–440 nm, with increments of 10 nm; (**c**) PL spectra of CQDs (1:0.5), CQDs (1:1), CQDs (1:1.5) and CQDs (1:2) in aqueous solutions (The inset is an enlarged view of emission area in the range of 440 nm–550 nm); (**d**) PL decay curves of CQDs under excitation wavelength of 400 nm.

In order to clarify the influence mechanism of reactants ratio on the optical properties of N, Cl co-doped CQDs, their absorption and photoluminescence properties have been studied in detail. Figure 7a displays the ultraviolet-visible (UV-Vis) absorption spectra of CQDs (1:0.5), CQDs (1:1), CQDs (1:1.5) and CQDs (1:2) samples. All spectra exhibit three absorption peaks located at ~225nm, ~260 nm and ~292 nm, which corresponds to π-π* transition of C=C bond, C=N bond and Cl-related state in N, Cl co-doped CQDs, respectively [80,81]. Obviously, the absorption spectra exhibit great dependence on the reactants ratio of CA:BD. For the absorption peak located at 225 nm, as compared to CQDs (1:0.5), a slight hyperchromic effect can be observed in CQDs (1:1) and then turns into obvious hypochromic effect for CQDs (1:1.5) and CQDs (1:2) samples. The hyperchromic effect is probably related to the increased conjugate π-domain due to adding more BD in the reaction. Once the reagent of BD continues to increase, the enlarged particle size of CQDs reduced the distance between CQDs (as shown in Figure 2), so that the π–π interaction can be effectively occurred, which finally resulted in hypochromic effect [82,83]. Moreover, Noor-Ul-Ain reported doping chlorine into CQDs would introduce additional energy level between the C_π_-C_π_*, called the Cl-related states [84]. The corresponding absorption peak at 292 nm observed in our samples coincided with the calculated transition energy from C_π_ to Cl-related states [28,69]. The absorption intensity of 292 nm peak exhibits a similar variation tendency via reactants ratio of CA:BD in comparison with 225 nm absorption peak, implying Cl-related states might play a great role in the photoluminescence process. It is worth pointing out here that usually the C=O contributes to the luminescence process of CQDs [85,86]. As shown in the XPS and FTIR spectra above, a relative small content of C=O bond can be observed but a large number of C–O exists in the form of C–OH [78,79]. What needs to be explained is that the amount of C–OH is depending on the degree of dryness of the sample during the characterization, since the CQDs are stored in an aqueous solution, the aqueous solution contains a large amount of C–OH. According to the previous reports, the typical absorption peak of C=O are usually located at 300–400 nm [85,86]. However, no absorption peak at the region of 300–400 nm appeared in our case, implying that C–O/C=O did not play a leading role in the luminescence process of CQDs. 

Figure 7b shows the excitation wavelength-dependent photoluminescence (PL) spectra of CQDs (1:1). As shown in Figure 7b, a characteristic green emission at 512 nm and a shoulder blue emission at 466 nm can be observed in Figure 7b. The blue emission located at 466 nm is usually assigned to the conjugated π-domain including C=C or C=N [17,87]. Obviously, the green emission centered at ~512 nm is beyond emission wavelength region of conjugated π-domain [17,87]. According to the scheme image of synthetic process shown in Figure 1, only Cl exists in the CQDs beside C and N groups. Moreover, an absorption peak corresponding to Cl-related states appeared in Figure 7a. Therefore, it is reasonable to deduce that this main green emission in N, Cl co-doped CQDs is probably related to the Cl-states [28]. No excitation wavelength dependence for 466 nm emission indicated the good crystallization quality of CQDs, which confirms the results of TEM and Raman measurement. Furthermore, it can be seen from Figure 7b, when the excitation wavelength increases from 380 to 440 nm, the PL peaks exhibit slight excitation wavelength dependence from 512 to 522 nm. Such excitation-dependent PL behavior of the CQDs is similar to the previous reports [88,89,90]. Wu et al. reported that the excitation-dependent emission is associated with the surface defects resulted from C–OH and C=O groups in the CQDs [90]. These C–OH and C=O groups form the emissive traps present on the CQDs surface. At certain excitation wavelengths, some of these emissive sites would be excited and fluoresce, giving rise to the induced dependent spectra. As for our as-prepared CQDs, a lot of oxygen-containing groups on the surface of CQDs such as C–OH, C=O and COOH indeed testified by XPS and FTIR measurements. It is worth mentioning that, a portion of the C–OH bond is derived from the air during the XPS test and another part of the C–OH bond is attributed to the degree of dryness of the sample during the FTIR characterization, since the CQDs are stored in an aqueous solution. Some of these oxygen-containing groups could form “surface states” and results in excitation-dependent PL behavior of as-prepared CQDs. Figure 7c presents the PL spectra of CQDs (1:0.5), CQDs (1:1), CQDs (1:1.5) and CQDs (1:2) samples. The peak position of blue emission remained at 466 nm via tuning the ratio of CA:BD, further testifying that this emission was originated from the conjugated π-domain. The peak center of green emission for CQDs (1:0.5), CQDs (1:1), CQDs (1:1.5) and CQDs (1:2) are located at 507 nm, 511 nm, 507 nm and 491 nm, respectively. Obviously, this green emission firstly exhibits slight red-shift and then turns into blue-shift as shown in the inset of Figure 7c, which might be ascribed to the competition of chlorine-related states, quantum confinement effect (QCE) and conjugated π-domain. In detail, Bolton O. reported that red-shift takes place in the emission wavelength once halogen elements such as chlorine are introduced into CQDs [91]. Graphene is a single atomic layer of graphite with an infinite exciton Bohr radius due to its linear energy dispersion relation of the charge carriers, resulting in QCE for graphene of any finite size [92]. Meanwhile, under the function of QCE, increased size can increase the band gap to achieve red-shift of luminescence [93,94]. The above two reasons might be the reason for the red shift of CQDs (1:1) compared to the CQDs (1:0.5), since more Cl has been introduced into the CQDs and QCE start to work. But CQDs (1:2) exhibits a slight blue-shift compared to CQDs (1:0.5)~CQDs (1:1.5). Since the atom ratio of N:Cl is 3 in the BD molecular formula, the increase amplitude of nitrogen is much larger than that of chlorine in CQDs. Li et al. reported nitrogen atoms in CQDs could contribute to the blue-shift of luminescence center [95]. However, the size difference between CQDs (1:2) and CQDs (1:1) is only 0.8 nm. The QCE might be covered up by much more nitrogen introduction, so that a slight blue-shift was observed. Thus, it is reasonable for us to believe that due to the competition between the conjugated π-domain, quantum confinement effect and chlorine-related states, the green emissions for CQDs (1:1.5) and CQDs (1:2) exhibit obvious blue-shift in comparison to that of CQDs (1:1).

Fluorescence lifetime is important information to reveal the carrier behavior. Figure 7d presents the TRPL spectra of CQDs (1:0.5), CQDs (1:1) and CQDs (1:2) samples. All the PL decay curves of as-prepared CQDs can be well fitted by double-exponential function: I(t) = A_1_exp (−t/τ_1_) + A_2_exp (−t/τ_2_), where τ_1_ and τ_2_ are the time constants of the two radiation decay channels; A_1_ and A_2_ are the corresponding amplitudes. The involved lifetimes of τ_1_ and τ_2_ have been summarized in Table 2, which is analogous to other reported values [96]. The double-exponential function decay curves for with fast and slow decay components all the CQDs revealed that all of samples have two luminescence pathways. According to the previous reports, the emission originated from defect states showed a longer recombination lifetime than that from intrinsic states [97,98,99,100]. Thus, the longer lifetime of CQDs in our case was attributed to the chlorine-related states [101] and the shorter lifetime corresponds to the conjugated π-domains (intrinsic state). When the ratio of CA:BD changes from 1:0.5 to 1:2, the doping amount of nitrogen and chlorine are increasing at the same time. Meanwhile, the conjugated π-domains have also grown, which is confirmed by the TEM image. Thus, the lifetime of τ_1_, τ_2_ and average lifetime exhibit an increasing trend in Table 2.

### 3.2. Effects of Reaction Time

**Figure 8:**
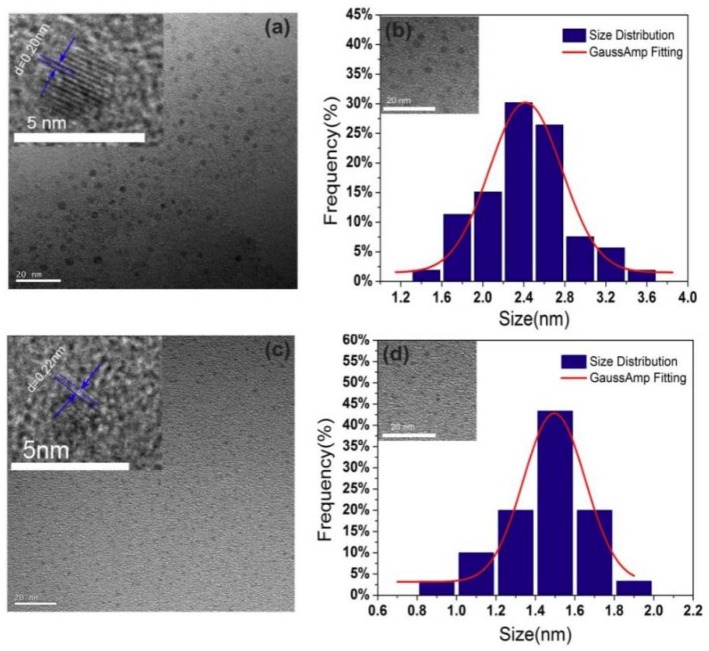
(**a**,**c**) TEM and HRTEM (the insert) images of CQDs (24 h) and CQDs (48 h); (**b**,**d**) The size distribution and local enlarged TEM image (inset) of the CQDs (24 h) and CQDs (48 h).

**Figure 9:**
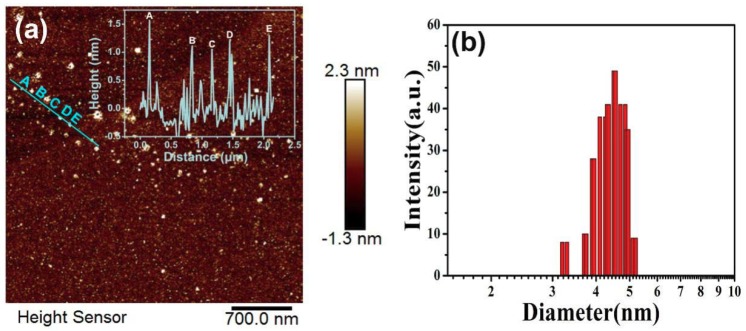
(**a**) AFM image of CQDs (48 h) deposited on a silicon slice (inset: the height profile along the line in the topographic image of the CQDs (48 h); (**b**) DLS image of CQDs (48 h).

To further reveal the effects of chlorine and nitrogen cooperation on the structure and optical properties of CQDs, the reaction time has been adjusted with keeping ratio as a constant of 1:1 and a set of samples of CQDs (6 h), CQDs (12 h), CQDs (24 h), CQDs (36 h) and CQDs (48 h) have been synthesized. The TEM and HRTEM images of CQDs (24 h) and CQDs (48 h) are shown in Figure 8. The description of TEM and HRTEM of CQDs (24 h) can be found in Figure 2a,b. As shown in Figure 8c,d, the as-prepared CQDs (48 h) is well-dispersed and the average particle size is ~1.5 nm, which is much smaller than those CQDs in recent reports [102,103]. The HRTEM image presented in the inset of Figure 8c shows the crystalline structure of CQDs (48h) and the distance between the lattice fringes is 0.22 nm, which is consistent to (100) facet of graphite [104]. Through comparing CQDs (24 h) with CQDs (48 h), a phenomenon can be clearly found that the increase of reaction time can result in a smaller particle size and larger lattice fringes. As the reaction time is prolonged, more opportunities can be provided for nitrogen and chlorine to enter into the carbon core. Since the ion radius of N (1.29 Å) and Cl (1.81 Å) is larger than that of C (0.86 Å), the lattice fringes of CQDs (48 h) are reasonable to be enlarged. In addition, the reduced size of CQDs (48 h) indicated that chlorine and nitrogen have an inhibition effect on the particle growth of CQDs [105]. The AFM and DLS technique was further utilized to demonstrate the topographic morphology and particle distribution of the CQDs (48 h), which was shown in Figure 9a,b. The typical topographic height of CQDs (48 h) is 0.5–1.5 nm, which indicated that the CQDs (48 h) consists of ca. 1–3 graphene layer. DLS further characterizes the distribution of CQDs in aqueous solution. The average particle size of the CQDs (48 h) is 4.48 nm. It is bigger than the TEM results, which is reasonable since DLS characterizes the particle size of the CQDs in the hydrated state.

**Figure 10:**
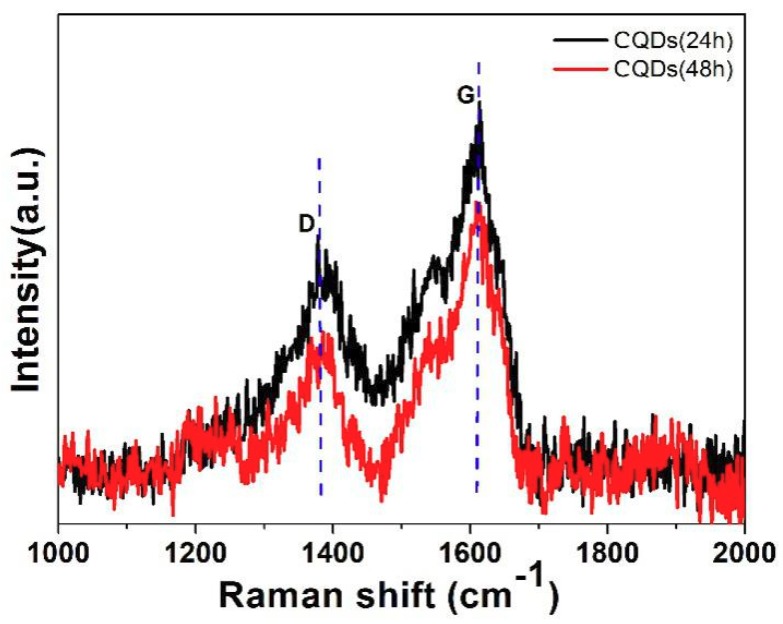
Raman spectra (λ_ex_ = 514 nm) of CQDs (24 h) and CQDs (48 h).

The Raman spectra (λ_ex_ = 514 nm) of CQDs (24 h) and CQDs (48 h) are shown in Figure 10. Two Raman vibrational peaks at 1382 cm^−1^ and 1611 cm^−1^ can be assigned to the defect-related D-band and G-band related to sp2 carbon networks. The intensity ratio of the D band and G band (I_D_/I_G_) is 0.52 and 0.62 for CQDs (24 h) and CQDs (48 h), respectively. The enlarged value of I_D_/I_G_ with prolonging the reaction time implied more defects appear in CQDs (48 h), indicating more nitrogen and chlorine have been incorporated into CQDs (48 h), which exhibits well agreement with the TEM results.

**Figure 11:**
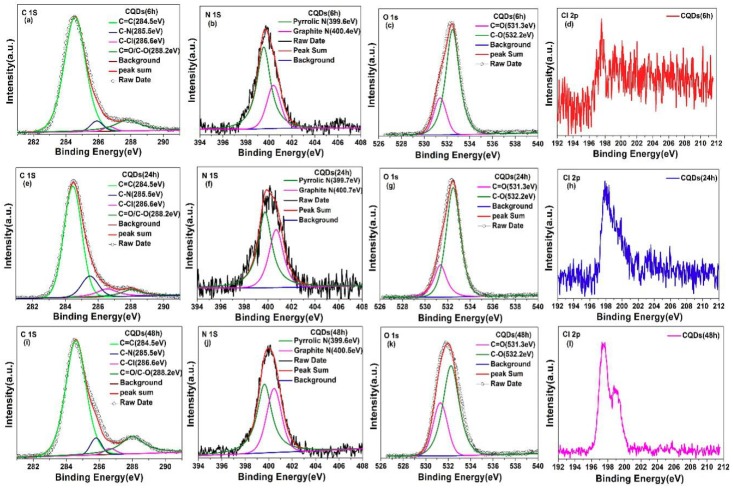
High-resolution C 1s, N 1s, O 1s and Cl 2p core level XPS spectra of CQDs (6 h) (**a**–**d**), CQDs (24 h) (**e**–**h**) and CQDs (48 h) (**i**–**l**).

The XPS technique was used to further investigate the effect of reaction time on the doping concentration of nitrogen and chlorine. The high-resolution XPS spectra of C 1s, N 1s, O 1s and Cl 2p for CQDs (6 h), CQDs (24 h) and CQDs (48 h) are shown in Figure 11. Similar to Figure 6, four fitting peaks corresponding to the carbon in C=C, C–N, C–Cl and C=O/C–O located at 284.5 eV, 285.5 eV, 286.6 eV and 288.2 eV, respectively. Based on Figure 11a,e,i, the percentages of C–N and C–Cl bonds in entire carbons for CQDs (6 h), CQDs (24 h) and CQDs (48 h) are listed in Table 3. Obviously, the content of chlorine and nitrogen in CQDs exhibits a slight increase as the reaction time was prolonged from 6 h to 48 h. As shown in Figure 11c,g,k, the two fitted peaks of the high-resolution O 1s spectrum at 531.3 and 532.4eV are ascribed to C=O and C–O groups, respectively. The C/O atomic ratio for the CQDs (6 h), CQDs (24 h) and CQDs (48 h) is 1.78, 1.49 and 2.5, respectively. It is worth mentioning that, hydrothermal reaction is carried out in a closed environment so that the content of the reactants is constant. When the reaction time is 6 h, the reaction is incomplete and less oxygen-containing groups attached on the surface of CQDs (6 h). With extending the reaction time to 24 h, more oxygen-containing functional groups owns enough time to attach on the surface of CQDs (24), so that the C/O atomic ratio of CQDs (24 h) is smaller than CQDs (6 h). Further prolonging the reaction time to 48 h, the CQDs can be purified to reduce some part of oxygen-containing functional groups on the surface of the CQDs (48 h), so that the C/O atomic ratio of CQDs (48 h) turned larger again. The high-resolution Cl 2p and N 1s core level XPS spectra were further presented in Figure 11 to characterize the doping profiles of chlorine and nitrogen. The Cl 2p^1/2^, Cl 2p^3/2^, pyrrolic N and graphitic N are located at 198 eV, 202 eV, ~399.4 eV and ~400.4 eV, respectively. All these results testified that nitrogen and chlorine has been successfully introduced into CQDs. The signal intensity of Cl 2p spectra continuously increases and the shape of peak gradually turns better, indicating that the doping concentration of chlorine increases. Meanwhile, the intensity ratio of graphitic N to pyrrolic N increased with prolonging the reaction time, which is reasonable since the CQDs can be continuously purified during the long hydrothermal process. Moreover, long reaction time could make more chlorine and nitrogen entered the crystal lattice of CQDs and influence on the carbonization of as-prepared CQDs at the same time, which is consistent with the Raman results.

**Figure 12:**
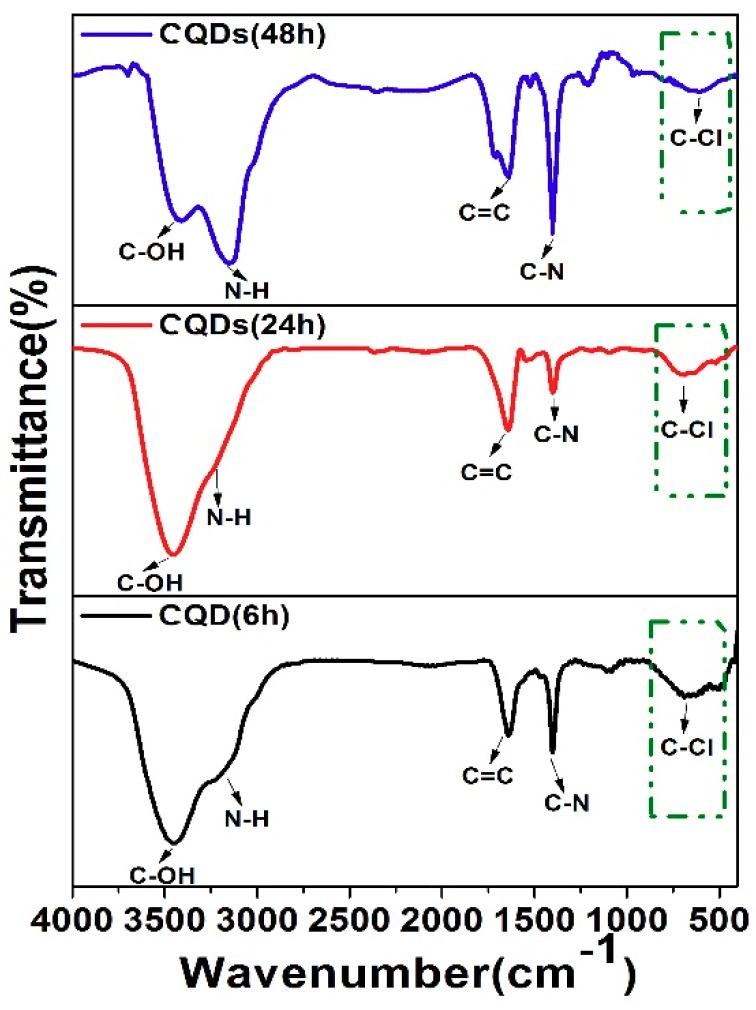
FTIR spectra of CQDs (6 h), CQDs (24 h) and CQDs (48 h).

The FTIR spectra of CQDs (6 h), CQDs (24 h) and CQDs (48 h) are shown in Figure 12. The characteristic absorption peak at 1640 cm^−1^, 1400 cm^−1^, 600–800 cm^−1^ and 3000–3600 cm^−1^ are attributed to stretching vibrations of C=C, C–N, C–Cl, C–OH and N–H bond [19,106], respectively. With prolonging the reaction time, the intensities of peaks related to N and Cl exhibit enhancement, indicating more N and Cl incorporates into the CQDs, at the same time, the carbon core is a purification process, so the C=C bond has a slight increase in the FTIR spectra, which has a good agreement with the above Raman and XPS results. It is noteworthy that the signal of C=O bond almost disappeared, which did not contribute to the luminescence process of CQDs. 

**Figure 13:**
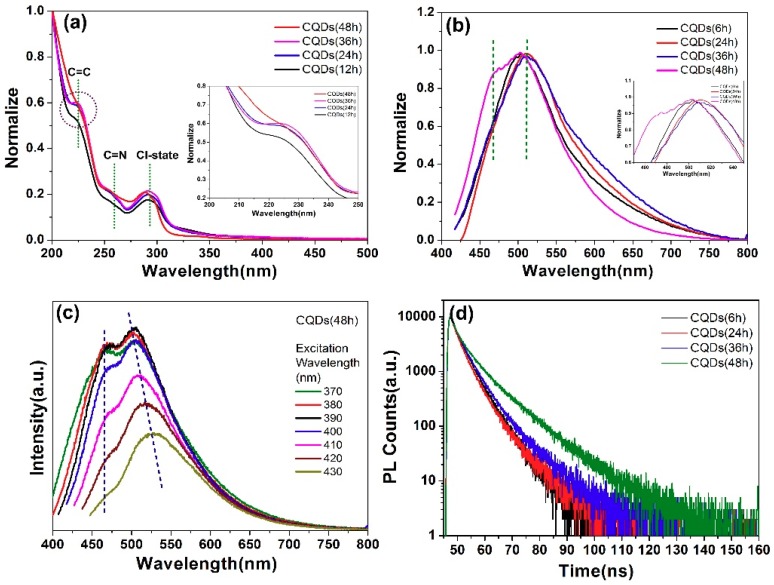
(**a**) UV-Vis absorption spectra of CQDs (12 h), CQDs (24 h), CQDs (36 h) and CQDs (48 h) in aqueous solutions (The inset is an enlarged view of absorbed by C=C); (**b**) PL spectra of CQDs (6 h), CQDs (24 h), CQDs (36 h) and CQDs (48 h) in aqueous solutions (The inset is an enlarged view of emission area in the range of 450 nm–550 nm); (**c**) PL spectra of CQDs (48 h) excited at wavelength of 370–430 nm, with increments of 10 nm; (**d**) PL decay curves of CQDs under excitation wavelength of 400 nm.

Figure 13a shows the absorption spectra of CQDs (12 h), CQDs (24 h), CQDs (36 h) and CQDs (48 h). Two characteristic peaks at ~225 nm, ~260 nm and ~292 nm can be ascribed to the π-π* transition of C=C, C=N bond and Cl-related state between π valence band and π* conduction band [63,69] respectively. Similar to Figure 7a, no absorption peak of C=O was obtained, which is consistent to the results of Figure 12. The peak intensity of CQDs (12 h) at ~225 nm is pretty weak, which was mainly caused by the incomplete formation of carbon core due to the short reaction time. With prolonging the reaction time to 36 h, the peak intensity of ~225 nm gradually enhanced, indicating the amount of π-domain increase step by step. However, when the reaction time is prolonged to 48 h, the peak intensity of ~225 nm starts to decrease due to more nitrogen or chlorine entering into the π-conjugated area. The intensity of peak located at ~260 nm and ~292 nm enhanced slightly during prolonging the reaction time to 48 hours, indicating that more N, Cl-related states existed in CQDs (48 h).

Figure 13b shows the PL spectra of CQDs (6 h), CQDs (24 h), CQDs (36 h) and CQDs (48 h). As shown in Figure 11b, the main luminescence center related to Cl-states of CQDs (6 h), CQDs (24 h), CQDs (36 h) and CQDs (48 h) located at 503 nm, 511 nm, 511 nm and 502 nm under excitation of 400 ex, respectively. With prolonging the reaction time from 6 h to 36 h, the emission peak position exhibits a slight red-shift due to more Cl incorporating into CQDs [91]. A blue-shift can then be observed when reacting for 48 h. As revealed by TEM results, the size of CQDs (48 h) (1.5 nm) is smaller than that of CQDs (24 h) (2.4 nm). Such-blue shift can be assigned to the quantum confinement effects. Moreover, the shoulder emission peak at 466 nm derived from the conjugated π-domain obviously appeared after the reaction time reached to 48 h [107,108]. It is well known that the reacting duration is a purification process for the CQDs. The degree of carbonization in CQDs (48 h) is better than CQDs (6–36 h) proved by the above Raman results, which is beneficial for the appearance of shoulder peak at 466 nm. Figure 13c displays the PL spectra of CQDs (48 h) at different excitation wavelength. A slight excitation dependent emission with the maximum emission at 512 nm due to the oxygen-containing groups on the surface of CQDs such as C–OH, C=O and COOH and independent emission at 466 nm attributed to the good crystallization quality of CQDs confirmed by TEM and Raman results, which is similar to Figure 7b. The TRPL spectra of CQDs (6 h), CQDs (24 h), CQDs (36 h) and CQDs (48 h) are shown in Figure 13d. The derived lifetimes of τ_1_ and τ_2_ by double-exponential function fitting have been summarized in Table 4, which is analogous to other reported values [106]. As similar as Figure 7d, the longer lifetime of CQDs could attribute to the chlorine-related states and the shorter lifetime corresponded to the conjugated π-domains (intrinsic state). The extended average lifetime was attributed to the purification of carbon core and amount of nitrogen/chlorine in CQDs. The longer lifetime exhibited an increasing trend since more chlorine was introduced into the CQDs. 

In order to further explore the fluorescence properties of the as-prepared CQDs, the quantum yield (QY) of the CQDs was investigated. The QY of the CQDs (24 h) were investigated. The QY of CQDs (24 h) was calculated with the following equation:

$$Q=Q_{R}\frac{I}{I_{A}}\frac{A_{R}}{A}\frac{n^{2}}{n_{R}{}^{2}}$$

where Q is the quantum yield, I is the measured integrated emission intensity, n is the refractive index and A is the optical density. The subscript R refers to the reference fluorophore of known quantum yield. Under the excitation of 380 nm, the QY of the CQDs (24 h) was measured to be 10.12% using quinine sulfate in 0.1 M H_2_SO_4_ (QY = 0.54) as a reference.

**Figure 14:**
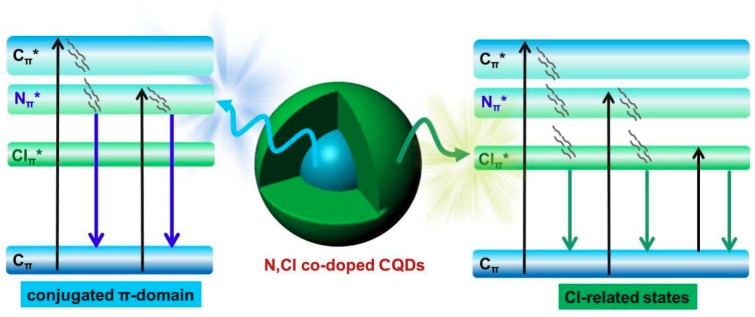
The speculated energy level diagram of the nitrogen and chlorine co-doped CQDs.

At present, the PL mechanism of carbon quantum dots is still an open topic, which needs deeper exploration. In the typical PL mechanism, it can be divided into two major categories: one is intrinsic state related to conjugated π-domains in CQDs [92,109], the other is surface/defect/edge state related to the surface groups or defects [15]. Based on the above massive data analysis, a novel PL mechanism has been proposed. As shown in Figure 14, the emission in blue-green regions of as-prepared CQDs can attribute to direct recombination of excited electrons from the conjugated π-domain and Cl-related state. The chlorine and nitrogen could introduce additional energy level between C_π_ and C_π*_. Based on the Figure 6, the electron transitions can happen from HOMO (C_π_) to LUMO (Cl_π*_), LUMO (N_π*_) and LUMO (C_π*_). Due to nonradiative processes such as vibration relaxation and so forth, the excited electrons located at LUMO (C_π*_) level will relax to LUMO (Cl_π*_) or LUMO (N_π*_), thus two emissions at 466 nm derived from the conjugated π-domain and 512 nm originated from chlorine-related states happens at the same time. With variation of nitrogen and chlorine doping concentration, these two emissions will be tuned. Thus, the synergy and competition mechanism of chlorine-states and conjugated π-domain play the key role in the luminescence process of nitrogen and chlorine co-doped CQDs.

## 4. Conclusions

In summary, by nitrogen and chlorine co-doping, we successfully tune the emission wavelength of CQDs and further investigate co-doping effects on the luminescent properties of CQDs. We succeed to invent the ultra-small nitrogen and chlorine co-doped CQDs with unique blue-green double emissions by a facile hydrothermal method. The blue emission at 466 nm derived from the conjugated π-domain and green emission at 512 nm originated from chlorine-related states. It is revealed that chlorine and nitrogen introduced new energy levels between C_π_ and C_π_* and the tunable blue-green double emissions of nitrogen and chlorine co-doped CQDs attribute to the synergy and competition mechanism of conjugated π-domain, quantum confinement effect and Cl-related state, which can be realized by controlling mole ratio of citric acid to BD and reaction time simply. The quantum yield of as-prepared CQDs is 10.12%, which is lower than other traditional semiconductor quantum dots such as CdSe and PbS quantum dots but it is low in toxicity and super water-soluble, which is beneficial for bio-application. To enhance the luminescence quantum yield of CQDs, the works on removing the surface functional groups of CQDs, increasing the degree of carbonization of CQDs, or regulating the particle size and soon on are necessary to be carried out in the future.

## Figures and Tables

**Table 1:** Intensity proportion of C–N and C–Cl in the C 1s spectra of CQDs (1:0.5), CQDs (1:1), CQDs (1:1.5) and CQDs (1:2).

Sample	CQDs (1:0.5)	CQDs (1:1)	CQDs (1:1.5)	CQDs (1:2)
**C–N**	2.92%	4.46%	6.42%	10.96%
**C–Cl**	1.08%	1.94%	2.86%	4.10%

**Table 2:** The lifetime components of CQDs (1:0.5), CQDs (1:1) and CQDs (1:2).

Sample	τ_1_ (ns)	τ_2_ (ns)	τ_average_ (ns)	χ^2^
**CQDs (1:0.5)**	2.21	5.56	2.75	0.93
**CQDs (1:1)**	2.46	6.11	3.32	1.05
**CQDs (1:2)**	3.08	7.97	4.23	0.86

**Table 3:** Intensity proportion of C–N and C–Cl in the C1s spectra of CQDs (6 h), CQDs (24 h) and CQDs (48 h).

Sample	CQDs (6 h)	CQDs (24 h)	CQDs (48 h)
C-N	4.13%	4.46%	5.40%
C-Cl	1.57%	1.94%	2.16%

**Table 4:** The lifetime components of CQDs (12 h), CQDs (24 h), CQDs (36 h) and CQDs (48 h).

Sample	τ_1_ (ns)	τ_2_ (ns)	τ_average_ (ns)	χ^2^
**CQDs (6 h)**	2.31	5.29	3.57	1.54
**CQDs (24 h)**	2.46	6.11	3.32	1.05
**CQDs (36 h)**	2.90	7.11	3.94	0.78
**CQDs (48 h)**	2.50	9.47	4.62	0.68
